# Fatty acid dysregulation in asthma: linking immunometabolic remodeling, phenotypic heterogeneity, and metabolic precision medicine

**DOI:** 10.3389/fimmu.2026.1974223

**Published:** 2026-10-19

**Authors:** Yuan Yuan, Yunhao Peng

**Affiliations:** 1 Department of Respiratory and Critical Care Medicine, Second Affiliated Hospital of Naval Medical University, Shanghai, China; 2 Sichuan University, Chengdu, China

**Keywords:** airway remodeling, asthma, fatty acid metabolism, immunometabolism, lipid dysregulation, precision medicine

## Abstract

Asthma is a highly heterogeneous chronic airway disease characterized by diverse inflammatory endotypes, variable airway remodeling patterns, and differential therapeutic responses. Although current classifications mainly rely on immune phenotypes, they do not fully explain severe steroid resistance, obesity-associated asthma, and inflammation-independent structural remodeling. Immunometabolic dysregulation, particularly fatty acid metabolic imbalance, has emerged as an additional dimension associated with asthma heterogeneity and the interplay among systemic metabolic status with airway inflammation and remodeling. In this review, we summarize major features of fatty acid dysregulation in asthma, including altered circulating and airway lipid profiles, PUFA-derived mediator imbalance, SFA-associated metabolic stress, and cell-type-specific alterations in fatty acid uptake, synthesis, oxidation, and storage. These alterations may affect airway epithelial cells, macrophages, lymphocytes, airway smooth muscle cells, and fibroblasts, thereby influencing epithelial barrier integrity, inflammatory programming, smooth muscle remodeling, and extracellular matrix deposition. Human studies have identified phenotype-associated lipid signatures, including AA-derived inflammatory mediators such as cysteinyl leukotrienes and PGD2 metabolites in T2-high asthma and alterations in linoleic acid and glycerophospholipid metabolism across granulocytic phenotypes, whereas obesity-associated asthma additionally reflects systemic metabolic disturbances arising from the adipose–lung axis. Much of the current human evidence remains associative, whereas functional support for specific fatty acids, lipid mediators, and metabolic pathways is derived largely from experimental and ex vivo studies. We further present a conceptual framework in which metabolic, inflammatory, and structural alterations may interact to contribute to persistent airway remodeling. Finally, we discuss the emerging translational potential of lipidomic-guided phenotyping and metabolism-directed interventions, while emphasizing that these approaches remain investigational and require prospective clinical validation.

## 1 Introduction

Asthma is a highly heterogeneous chronic airway disorder characterized by distinct clinical phenotypes and underlying molecular endotypes. Patients experiencing similar respiratory symptoms often exhibit significant variations in baseline inflammatory archetypes, the severity of airway remodeling, long-term disease progression, and therapeutic outcomes (1, 2). For decades, clinical stratification has predominantly relied on the dichotomy of Type 2 (T2)-high and T2-low immune responses. The conventional T2-high paradigm is driven by adaptive Th2 cells and group 2 innate lymphoid cells (ILC2s), which orchestrate the secretion of canonical interleukins, leading to elevated fractional exhaled nitric oxide (FeNO), immunoglobulin E (IgE) synthesis, and eosinophilic infiltration (3).

However, this traditional inflammatory classification fails to comprehensively address the broader spectrum of asthma phenotypes. Clinical evidence highlights four primary limitations of this framework:

- a substantial cohort of patients lacks classic T2 inflammatory hallmarks but presents with intractable, persistent symptoms (4);
- non-eosinophilic variants—specifically neutrophilic asthma and paucigranulocytic asthma—are poorly captured by traditional T2 biomarkers, yet they represent highly morbid clinical presentations (5);
- a spatial and temporal mismatch frequently exists between conventional systemic inflammatory indices and structural damage, as progressive subepithelial fibrosis can occur despite apparent biomarker control (6);
- and a significant subset of patients demonstrates insensitivity to mainstream anti-inflammatory regimens, most notably inhaled corticosteroids (ICS) (7).

Consequently, relying solely on canonical cytokine cascades and leukocyte counts is insufficient to resolve the pathogenic complexity of the disease. These unresolved features—including discordance between inflammatory biomarkers and structural remodeling, corticosteroid insensitivity, and the metabolic burden associated with obesity—suggest that additional biological processes beyond conventional immune classification may contribute to asthma heterogeneity.

Immunometabolism provides a robust framework to decode such complex chronic inflammatory diseases by directly linking cellular bioenergetics to immune cell fate and tissue homeostasis (8). Within this context, fatty acid dysregulation has emerged as a potentially important metabolic dimension associated with asthma pathophysiology, because fatty acids participate not only in systemic metabolic homeostasis but also in cellular energy metabolism, lipid mediator generation, oxidative stress responses, and structural-cell remodeling (9). Variations in localized fatty acid profiles, shifting ratios between distinct lipid classes, and intracellular metabolic rerouting—such as the deviation from mitochondrial beta-oxidation toward lipid droplet accumulation or the synthesis of lipotoxic species in lung resident immune and structural cells—may influence the airway microenvironment (8). Altered fatty acid profiles may serve as metabolic signatures of disease-associated lipid remodeling, whereas specific fatty acids and their downstream bioactive mediators may modulate cellular signaling and inflammatory responses. Specifically, they may influence the biophysical integrity of the epithelial barrier, alter the antigen-presenting maturation of dendritic cells, dictate helper T-cell differentiation pathways, drive macrophage polarization, and govern the phenotypic transition of structural mesenchymal cells (2, 10). Incorporating fatty acid dysregulation as a complementary metabolic axis may help explain aspects of asthma that are incompletely captured by conventional inflammatory metrics, including metabolic influences on non-T2 neutrophilic inflammation, corticosteroid insensitivity, obesity-associated disease, and structural remodeling that may persist despite apparent inflammatory control. Thus, evaluating these metabolic perturbations may provide an additional perspective for understanding the mechanistic divergence of asthma endotypes.

To reconcile these insights, this review systematically synthesizes the role of fatty acid dysregulation in asthma, with particular emphasis on whether metabolic alterations may help explain inflammatory heterogeneity, obesity-associated disease, corticosteroid insensitivity, and airway remodeling beyond conventional immune classification. We map the systemic and localized origins of these lipid imbalances and examine the molecular pathways through which they may influence the interconnected airway immune-structural network. Finally, we discuss the emerging translational potential of lipidomics-based stratification and phenotype-matched metabolic interventions.

## 2 Fatty acid dysregulation in asthma: sources and molecular landscape

### 2.1 Altered lipid landscape and conceptual framework of fatty acid dysregulation in asthma

Asthma is increasingly recognized as a disease accompanied by metabolic alterations, including disturbances in lipid and fatty acid metabolism. Recent metabolomic and lipidomic studies have revealed alterations in circulating lipid profiles and airway-localized lipid signatures in patients with asthma, while cellular studies have identified additional changes in intracellular fatty acid metabolic pathways. In this review, fatty acid dysregulation refers to coordinated but distinct alterations in fatty acid composition and availability, intracellular fatty acid uptake, synthesis, oxidation, storage, and the generation of downstream bioactive lipid mediators. Precursor fatty acids should therefore be distinguished from downstream lipid mediators, and circulating or airway lipid profiles should be distinguished from intracellular metabolic programs. These findings indicate that lipid remodeling is an important component of asthma biology and provides additional information beyond conventional inflammatory classification (11, 12).

Airway-based multi-omic analyses further demonstrate that metabolic changes occur within the pulmonary microenvironment. Altered lipid-related pathways identified in airway samples suggest that local metabolic remodeling is involved in asthma pathogenesis (13). Together, these studies establish lipid remodeling as an important metabolic feature of asthma.

### 2.2 Systemic and local sources of fatty acid imbalance

Fatty acid abnormalities in asthma arise from both systemic metabolic disturbances and local airway alterations. Obesity represents a major systemic condition associated with altered lipid metabolism. Dysfunctional adipose tissue promotes excessive lipid release, including increased circulating free fatty acids (FFAs), and contributes to the metabolic environment observed in obesity-related asthma (2).

However, circulating lipid changes alone cannot fully explain airway metabolic alterations. The lung possesses independent metabolic characteristics that can be influenced by allergens, environmental exposures, and chronic inflammatory conditions. Recent airway multi-omic studies suggest that local lipid remodeling may occur within the respiratory tract and differ from systemic metabolic profiles (13). Conceptually, circulating FFAs reflect systemic metabolic status, airway lipid mediators indicate local respiratory microenvironmental changes, and intracellular fatty acid handling represents cell-specific metabolic programming.

Therefore, asthma-associated fatty acid dysregulation reflects the combined effects of systemic metabolic status and airway-specific metabolic changes.

### 2.3 Precursor fatty acids and downstream bioactive lipid mediators

Precursor fatty acids and their downstream bioactive lipid mediators represent related but distinct levels of lipid biology. Omega-6 polyunsaturated fatty acids (n-6 PUFAs), particularly arachidonic acid (AA), serve as precursors for multiple lipid mediators involved in inflammatory signaling. Recent studies have demonstrated that abnormal AA oxidation generates bioactive lipid products enriched in severe asthma, highlighting the importance of lipid mediator dysregulation in advanced disease (14).

In contrast, n-3 PUFAs generate specialized pro-resolving mediators (SPMs), including resolvins and protectins, which participate in maintaining inflammatory balance. Recent studies have investigated alterations in n-3 PUFA-derived mediators in asthma-related conditions, suggesting that impaired lipid mediator balance may contribute to persistent airway inflammation (10). Beyond lipid mediator generation, fatty acid metabolism is also regulated by upstream metabolic pathways, in which glycolysis-derived acetyl-CoA serves as a central precursor for fatty acid and cholesterol synthesis, processes largely controlled by the sterol regulatory element-binding protein (SREBP) pathway (8).

Overall, asthma-associated fatty acid abnormalities involve coordinated changes among multiple lipid classes rather than alterations of a single fatty acid species.

## 3 Fatty acid-driven immunometabolic remodeling and signaling networks in asthma

### 3.1 Airway epithelial metabolic stress and lipid-mediated inflammatory signaling

Airway epithelial cells represent the first barrier exposed to environmental stimuli and function as active metabolic sensors that integrate external challenges with inflammatory responses. Beyond their structural role, epithelial cells undergo metabolic adaptation in response to allergens, oxidative stress, and inflammatory cytokines, thereby potentially influencing airway immune activation. Recent single-cell transcriptomic studies have demonstrated substantial heterogeneity among pulmonary epithelial populations, revealing distinct epithelial states associated with stress responses, immune regulation, and metabolic adaptation in healthy and diseased lungs (15, 16).

Lipid metabolic alterations represent an important component of epithelial metabolic remodeling. Asthma-associated lipidomic studies have revealed changes in fatty acid-derived metabolites and bioactive lipid mediator profiles, suggesting an association between disrupted lipid homeostasis and airway inflammation (17). Among these metabolites, oxidized fatty acid derivatives include linoleic acid-derived hydroxyoctadecadienoic acids (HODEs) and arachidonic acid-derived hydroxyeicosatetraenoic acids (HETEs). In nasal epithelial samples, 13-HODE has been reported as one of the most abundant detectable lipid mediators, and lipid mediator profiles differ by asthma status (18). In addition, metabolomic endotyping studies have identified asthma-associated metabolic subgroups with distinct lung-function profiles, supporting the broader relevance of metabolomic stratification in asthma (19). In asthma, altered AA-derived HETE profiles have been associated with disease-related metabolic and inflammatory changes (20). Altered profiles of these oxidized lipid mediators may therefore represent a potential association between abnormal fatty acid metabolism and epithelial inflammatory activation.

Mitochondrial metabolism may represent another interface connecting fatty acid dysregulation with epithelial dysfunction. Fatty acids serve as substrates for mitochondrial β-oxidation and energy production; consistent with this concept, CPT1A deficiency in airway epithelial cells has been linked to impaired mitochondrial homeostasis, increased oxidative stress, and epithelial barrier dysfunction in severe asthma (21). Mitochondrial reactive oxygen species (ROS) function not only as indicators of metabolic stress but also as signaling molecules regulating inflammatory pathways and cellular adaptation. The nuclear factor erythroid 2-related factor 2 (NRF2) pathway represents a major antioxidant response system integrating redox regulation with intermediary metabolism. Although NRF2 activation supports cellular antioxidant and cytoprotective responses, persistent metabolic stress may exceed antioxidant capacity and contribute to chronic inflammatory signaling (22).

Together, these findings suggest that epithelial lipid metabolic remodeling may participate in the interplay among environmental stress, metabolic dysfunction, and inflammatory amplification. However, the contribution of specific lipid metabolic pathways to distinct asthma phenotypes remains incompletely understood. Moreover, whether these metabolic alterations function as upstream drivers of epithelial inflammation or arise secondarily from the chronic inflammatory environment remains to be further explored.

### 3.2 Macrophage lipid sensing, fatty acid oxidation and inflammatory programming

Macrophages are highly plastic immune cells whose functional states are strongly influenced by metabolic conditions within the airway microenvironment. Fatty acid uptake, intracellular lipid trafficking, mitochondrial fatty acid oxidation (FAO), and lipid mediator production collectively contribute to macrophage adaptation and inflammatory responses.

Lipid sensing represents an initial mechanism linking extracellular fatty acid availability with macrophage function. CD36, a scavenger receptor involved in fatty acid uptake and lipid internalization, has been shown in metabolic and macrophage inflammation models to regulate intracellular lipid accumulation and macrophage metabolic adaptation (23, 24). In addition, fatty acid-binding proteins (FABPs) function as intracellular lipid chaperones that facilitate fatty acid transport and regulate lipid-responsive signaling pathways (25).

Among FABP family members, FABP5 has emerged as a potential metabolic regulator connecting intracellular fatty acid handling with macrophage inflammatory responses. FABP5 has been reported to regulate lipid trafficking and may interact with lipid-responsive transcriptional programs, including PPARγ-associated pathways. Through regulation of fatty acid uptake, storage, and oxidation-related genes, PPARγ may function as a metabolic checkpoint controlling macrophage phenotype and inflammatory adaptation (26, 27).

The balance between fatty acid oxidation and lipid accumulation represents a major determinant of macrophage metabolic programming. At a broader immunometabolic level, AMPK acts as an energy sensor that promotes mitochondrial oxidative metabolism and maintains metabolic flexibility during cellular stress (28). Conversely, nutrient-rich inflammatory environments activate mTOR-dependent anabolic programs, promoting lipid synthesis and metabolic remodeling (29). Therefore, macrophage function may be influenced by the dynamic balance between catabolic pathways supporting FAO and anabolic pathways promoting lipid accumulation. And the asthma-specific relevance of this balance requires further validation.

Abnormal lipid accumulation, particularly saturated fatty acid enrichment, may promote macrophage inflammatory activation through cellular stress responses and inflammasome pathways. Evidence from obesity-related metabolic inflammation and broader lipid-stress models suggests that saturated fatty acid accumulation and lipid overload may activate the NLRP3 inflammasome, resulting in increased IL-1β production and inflammatory amplification (30, 31). Whether this SFA–organelle stress–NLRP3 axis operates directly in airway macrophages during obese or non-type 2 asthma requires further validation.

Single-cell studies have further revealed substantial heterogeneity among pulmonary macrophage populations, including tissue-resident alveolar macrophages and recruited monocyte-derived macrophages. These populations exhibit distinct transcriptional and metabolic signatures, suggesting that fatty acid metabolic regulation may contribute to macrophage specialization in different inflammatory contexts (32, 33).

### 3.3 Lymphocyte metabolic reprogramming and inflammatory phenotype determination

Adaptive immune cells undergo extensive metabolic remodeling during activation and differentiation. Distinct metabolic programs regulate lymphocyte proliferation, cytokine production, and lineage commitment. Activated effector T cells generally rely on anabolic metabolism, whereas regulatory populations preferentially utilize oxidative pathways to maintain functional stability (34, 35).

Studies in T-cell differentiation models indicate that Th17 cells exhibit a strong dependence on *de novo* fatty acid synthesis. Experimental studies have demonstrated that inhibition of fatty acid synthesis impairs Th17 differentiation while favoring regulatory T-cell development, indicating that lipid biosynthetic pathways function as critical metabolic checkpoints controlling inflammatory T-cell fate decisions (36, 37).

In contrast, regulatory T cells preferentially utilize mitochondrial oxidative phosphorylation and fatty acid oxidation to maintain suppressive functions. These findings provide mechanistic context for understanding how lipid biosynthetic and oxidative programs may influence inflammatory versus regulatory T-cell responses (38, 39).

mTOR and HIF-1α pathways integrate nutrient availability with T-cell differentiation. mTOR activation promotes effector T-cell programs, whereas HIF-1α-dependent metabolic regulation influences the reciprocal differentiation between Th17 cells and Tregs (40, 41).

Although most studies have focused on Th17 and Treg metabolism, emerging evidence suggests that lipid metabolic pathways may also influence type 2 immune responses. Th2 cells, which are central mediators of allergic asthma, require metabolic adaptation involving glycolysis, mTOR signaling, and lipid sensing pathways to sustain cytokine production. However, the specific contribution of fatty acid metabolism to Th2 differentiation remains less well defined compared with Th17 biology. In asthma, particularly severe and non-type 2 inflammatory phenotypes, Th17/IL-17-associated inflammation has been implicated in disease persistence and corticosteroid insensitivity, but direct evidence linking fatty acid metabolic rewiring in T cells to these asthma phenotypes remains limited. Increased Th17 responses and IL-17-associated inflammation have been implicated in severe asthma and corticosteroid-insensitive disease (42).

### 3.4 Fatty acid metabolism and airway remodeling

Airway remodeling encompasses complex structural alterations, including subepithelial fibrosis, extracellular matrix (ECM) deposition, epithelial–mesenchymal interactions, and airway smooth muscle (ASM) dysfunction. Metabolic reprogramming may contribute to the functional and activation states of structural cells involved in these chronic alterations, with the direction and functional consequences of fatty acid metabolism varying across cell types (43–45).

Fibroblast activation requires metabolic rewiring involving mitochondrial bioenergetics, lipid utilization, and biosynthetic pathways. These altered metabolic states promote the fibroblast-to-myofibroblast transition, enhance ECM deposition, and regulate profibrotic signaling cascades such as transforming growth factor-beta (TGF-β) (46, 47). Although these metabolic drivers have been predominantly established in pulmonary fibrosis models, they provide mechanistic context for fatty acid-driven airway remodeling in asthma.

Notably, ASM cells exhibit cell-type-specific metabolic remodeling in asthma. In contrast to the impaired FAO reported in airway epithelial cells, asthmatic bronchial smooth muscle (BSM) cells show a metabolic shift toward enhanced mitochondrial fatty acid β-oxidation, accompanied by increased fatty acid consumption and ATP production, which supports BSM cell proliferation and airway remodeling (48). Epithelial dysfunction may influence adjacent mesenchymal cells through epithelial–mesenchymal crosstalk, thereby contributing to fibroblast activation and airway remodeling. Fatty acid metabolic remodeling may intersect with this epithelial–mesenchymal signaling network, although direct evidence for this mechanism in asthma remains limited. **Figure 1** summarizes the integrated immunometabolic network through which fatty acid dysregulation influences epithelial, myeloid, and lymphoid compartments, highlighting how systemic and local lipid disturbances shape distinct inflammatory trajectories across asthma endotypes.

**Figure 1:**
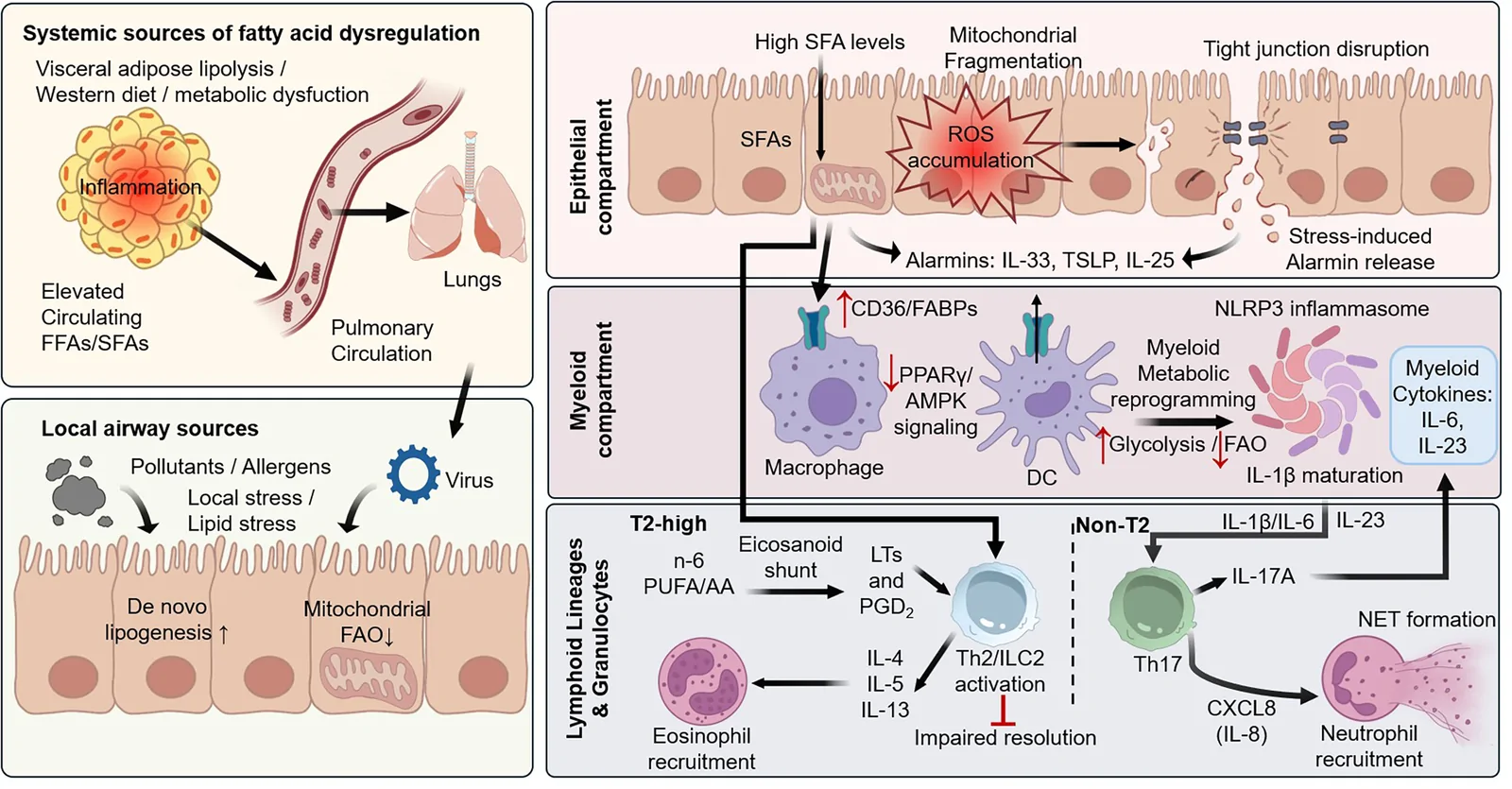
Fatty acid dysregulation shapes the epithelial–myeloid–lymphoid immunometabolic network in asthma. Systemic lipid spillover provides circulating metabolic signals, whereas local airway stress reshapes airway lipid mediator production and cell-intrinsic fatty acid metabolism. Elevated SFAs may induce or amplify epithelial mitochondrial stress, ROS accumulation, tight junction disruption, and alarmin release. In myeloid cells, increased CD36/FABP-mediated fatty acid uptake is associated with reduced PPARγ/AMPK signaling, metabolic reprogramming, NLRP3 activation, IL-1β maturation, and IL-6/IL-23 production. These signals promote divergent inflammatory trajectories: n-6 PUFA/AA-derived eicosanoids favor Th2/ILC2 activation and eosinophilic inflammation, whereas SFA-driven myeloid cytokines may favor Th17 activation, IL-17A production, neutrophil recruitment, and NET formation.

## 4 Fatty acid heterogeneity across asthma phenotypes

Fatty acid-related metabolic features show substantial heterogeneity across asthma phenotypes, reflecting the complex interplay among T2 inflammation, granulocytic patterns, obesity, and systemic metabolic status, with distinct inflammatory environments likely shaping lipid profiles while lipid alterations may also feedback to influence inflammatory trajectories.

### 4.1 Lipolytic cascades and resolution deficiencies in type 2-high endotypes

In T2-high asthma, alterations in fatty acid and lipid mediator metabolism have been associated with inflammatory activity and may modulate type 2 immune responses. Altered PUFA metabolism, including changes in omega-6-derived inflammatory lipid mediators and n-3-derived pro-resolving pathways, has been associated with a tissue environment characterized by enhanced type 2 inflammation and impaired resolution capacity (49). At the metabolic level, this lipid imbalance may redirect PUFA flux toward AA-derived enzymatic pathways, resulting in preferential generation of inflammatory eicosanoids that reinforce type 2 immune activation.

Crucially, a bidirectional interaction exists between metabolic disturbance and type 2 inflammatory signaling. In T2-high asthma, increased AA-derived lipid mediators, including cysteinyl leukotrienes and PGD2 metabolites, have been observed in clinical cohorts and are associated with type 2 inflammatory activity (50). PGD2 metabolites can further activate patient-derived eosinophils and ILC2s through DP2 signaling (51). Conversely, alterations in lipid resolution pathways may impair endogenous inflammation control, as reduced production or activity of SPMs, including lipoxins and resolvins, has been associated with persistent airway inflammation and defective resolution responses (52). Consistent with this concept, a recent longitudinal study of severe asthma identified a subgroup with persistently low plasma RvD1, greater sputum eosinophilia, enhanced T2 inflammation, lower lung function, and more frequent exacerbations (53). This imbalance between inflammatory lipid mediator generation and resolution capacity may create a metabolic-inflammatory feedback loop that contributes to the chronicity of T2-high asthma, suggesting that T2-associated lipid profiles may represent both consequences of type 2 inflammation and contributors to its persistence.

### 4.2 Divergent molecular architectures of non-T2 and obesity-related subtypes

Compared with classical T2-high asthma, non-T2 and obesity-related asthma are more frequently associated with neutrophilic inflammation, metabolic stress, and broader disturbances in cellular or systemic metabolism. However, these phenotypic categories are not mutually exclusive, and obesity-related asthma may display either T2-high or non-T2 inflammatory features (54). Non-T2 asthma encompasses heterogeneous inflammatory patterns, including neutrophilic and paucigranulocytic phenotypes (55). Epithelial and metabolic alterations have also been identified in asthma, with bronchial epithelial metabolic programs and airway metabolomic signatures varying according to inflammatory characteristics and disease severity (56). Metabolic dysfunction in airway immune and structural cells may further contribute to persistent inflammatory activation, with alterations in mitochondrial metabolism and cellular energy homeostasis providing a broader metabolic context for asthma (57, 58). Importantly, human sputum metabolomic profiling has demonstrated distinct metabolic signatures across eosinophilic, neutrophilic, and paucigranulocytic asthma phenotypes, including alterations in linoleic acid and glycerophospholipid metabolism, supporting their potential value as phenotype-associated metabolic signatures (56). These signatures may partly reflect phenotype-specific inflammatory environments, including epithelial stress, granulocytic inflammation, and innate immune activation. In particular, SFAs accumulation has been associated with activation of innate immune pathways, including TLR4 and NLRP3 inflammasome signaling and increased IL-1β production, while Th17/IL-17A-associated responses are implicated in neutrophilic inflammation and corticosteroid insensitivity in severe asthma (59, 60). This supports a possible feed-forward interaction between lipid stress and non-T2 inflammatory pathways, although direct causal evidence remains limited.

Obesity-related asthma represents systemic metabolic disturbances that may interact with airway inflammation through the adipose–lung axis. Dysfunctional adipose tissue in obesity releases metabolic and inflammatory signals, including altered adipokine profiles and circulating lipid mediators, which may contribute to pulmonary immune dysregulation and airway inflammation (61, 62). Importantly, these circulating metabolic signals represent systemic exposures, whereas the resulting changes in pulmonary macrophage polarization, airway lipid mediator production, and intracellular fatty acid handling represent local or cell-specific responses within the lung. Among obesity-associated metabolic signals, leptin may interact with T2 cytokine signaling; recent human and experimental evidence indicates that leptin can augment IL-13-induced eotaxin production and airway eosinophilia in obesity-associated T2 asthma (63). Experimental models further suggest that obesity-associated metabolic conditions can alter pulmonary macrophage polarization and inflammatory activity (64). Systemic metabolic status, T2 inflammatory activity, granulocytic phenotype, and airway-specific metabolic abnormalities represent interacting dimensions of asthma heterogeneity.

### 4.3 Metabolic and structural remodeling in paucigranulocytic asthma

In severe, steroid-insensitive asthma, metabolic and oxidative disturbances may contribute to persistent inflammation and therapeutic resistance. Increased oxidative stress has been implicated in corticosteroid insensitivity through impairment of HDAC2 activity and disruption of glucocorticoid receptor-mediated transcriptional repression, allowing persistent activation of inflammatory gene programs despite corticosteroid treatment (65). These alterations may contribute to the maintenance of steroid-insensitive networks, particularly those involving neutrophilic inflammation and Th17/IL-17A signaling.

A distinct clinical challenge is paucigranulocytic asthma (PGA), which is characterized by limited eosinophilic or neutrophilic inflammation despite persistent airflow limitation and airway hyperresponsiveness. Recent studies indicate that PGA is associated with abnormalities in airway structural cells, including altered airway smooth muscle function and fibroblast-mediated remodeling processes, suggesting that disease activity may persist through inflammation-independent mechanisms (66, 67). Metabolic reprogramming within structural compartments, including altered lipid utilization and increased biosynthetic activity, has been proposed as a potential mechanism linking cellular metabolic stress with extracellular matrix deposition and airway remodeling (48). Therefore, PGA may provide a useful framework for refining asthma classification by highlighting structural and metabolic abnormalities that are not fully captured by conventional granulocytic inflammatory phenotyping.

Overall, current evidence is strongest for phenotype-associated lipid signatures and functional effects of selected lipid mediators or metabolic pathways, whereas direct evidence that fatty acid dysregulation causally determines specific asthma phenotypes remains limited. Whether phenotype-specific lipid profiles arise mainly as inflammatory consequences or contribute to early endotype divergence remains an important knowledge gap.

## 5 Fatty acid dysregulation-mediated airway remodeling

### 5.1 Epithelial injury, mucus hypersecretion, and defective repair kinetics

The persistence of airway remodeling in asthma involves a progressive disruption of epithelial homeostasis, in which altered lipid metabolism represents an emerging regulatory mechanism. Chronic exposure to elevated SFAs, particularly palmitic acid, can induce epithelial metabolic stress and inflammatory activation, accompanied by metabolic reprogramming involving fatty acid β-oxidation and related lipid pathways (68). Reduced mitochondrial fatty acid oxidation capacity, including alterations in carnitine palmitoyltransferase 1A (CPT1A)-dependent metabolism, has been associated with increased mitochondrial ROS production and compromised epithelial barrier integrity in severe asthma (21).

Separately, oxidative stress induced by neutrophil elastase has been shown to activate epithelial endoplasmic reticulum stress pathways, including IRE1α/XBP1 signaling, thereby promoting mucin 5AC (MUC5AC) production and secretion (69). These epithelial abnormalities may further contribute to airway remodeling by influencing neighboring immune and mesenchymal compartments. Collectively, fatty acid dysregulation may represent an important metabolic contributor linking epithelial dysfunction with structural airway remodeling.

### 5.2 Airway smooth muscle dysfunction and airway hyperresponsiveness

ASM cells represent an active structural compartment through which lipid mediator signaling and metabolic remodeling contribute to airway hyperresponsiveness (AHR). Arachidonic acid-derived lipid mediators, including cysteinyl leukotrienes and prostaglandins, regulate ASM contraction through specific G-protein coupled receptors, enhancing calcium-dependent contractile responses and airway narrowing (50).

Beyond acute contraction, asthmatic ASM cells exhibit a metabolic shift toward enhanced mitochondrial fatty acid β-oxidation, which is associated with increased proliferative activity. Asthmatic bronchial smooth muscle cells show increased mitochondrial respiration, fatty acid consumption, and expression of CPT2, whereas inhibition of CPT2 or LDL receptor markedly reduces their proliferation (48). These findings suggest that enhanced FAO can support ASM proliferation and remodeling, underscoring the cell-type-specific nature of fatty acid metabolic reprogramming in asthma.

### 5.3 Fibroblast activation, collagen deposition, and subepithelial fibrosis

Subepithelial fibrosis, characterized by extracellular matrix (ECM) accumulation and basement membrane thickening, represents a major component of airway remodeling in asthma. Airway fibroblasts contribute directly to this process through fibroblast-to-myofibroblast transition (FMT), enhanced collagen production, and dysregulated matrix turnover regulated by TGF-β-dependent signaling pathways (50). Recent studies have demonstrated that asthmatic fibroblasts exhibit enhanced profibrotic activity, including increased production of collagen and fibronectin, together with altered MMP/TIMP balance, thereby promoting persistent airway remodeling (45).

### 5.4 The metabolic–inflammatory–mechanical vicious cycle in remodeling persistence

The chronic progression and structural refractoriness of severe asthma may be sustained by an integrated, feed-forward loop connecting metabolic disruption, inflammatory persistence, and altered tissue mechanics. This proposed self-sustaining circuit may help explain the persistence and progression of severe, steroid-insensitive asthma phenotypes. Fatty acid dysregulation may act as an upstream contributor within this feed-forward process, potentially contributing to epithelial injury, myeloid inflammasome activation, and mesenchymal myofibroblast differentiation. These cellular transformations may contribute to excessive ECM deposition, smooth muscle hypertrophy, and progressive tissue stiffening, which alters the physical architecture of the airway wall. Crucially, this proposed metabolic–inflammatory–mechanical feedback loop provides a conceptual framework linking fatty acid dysregulation with persistent airway remodeling and severe asthma phenotypes, as illustrated in **Figure 2**.

**Figure 2:**
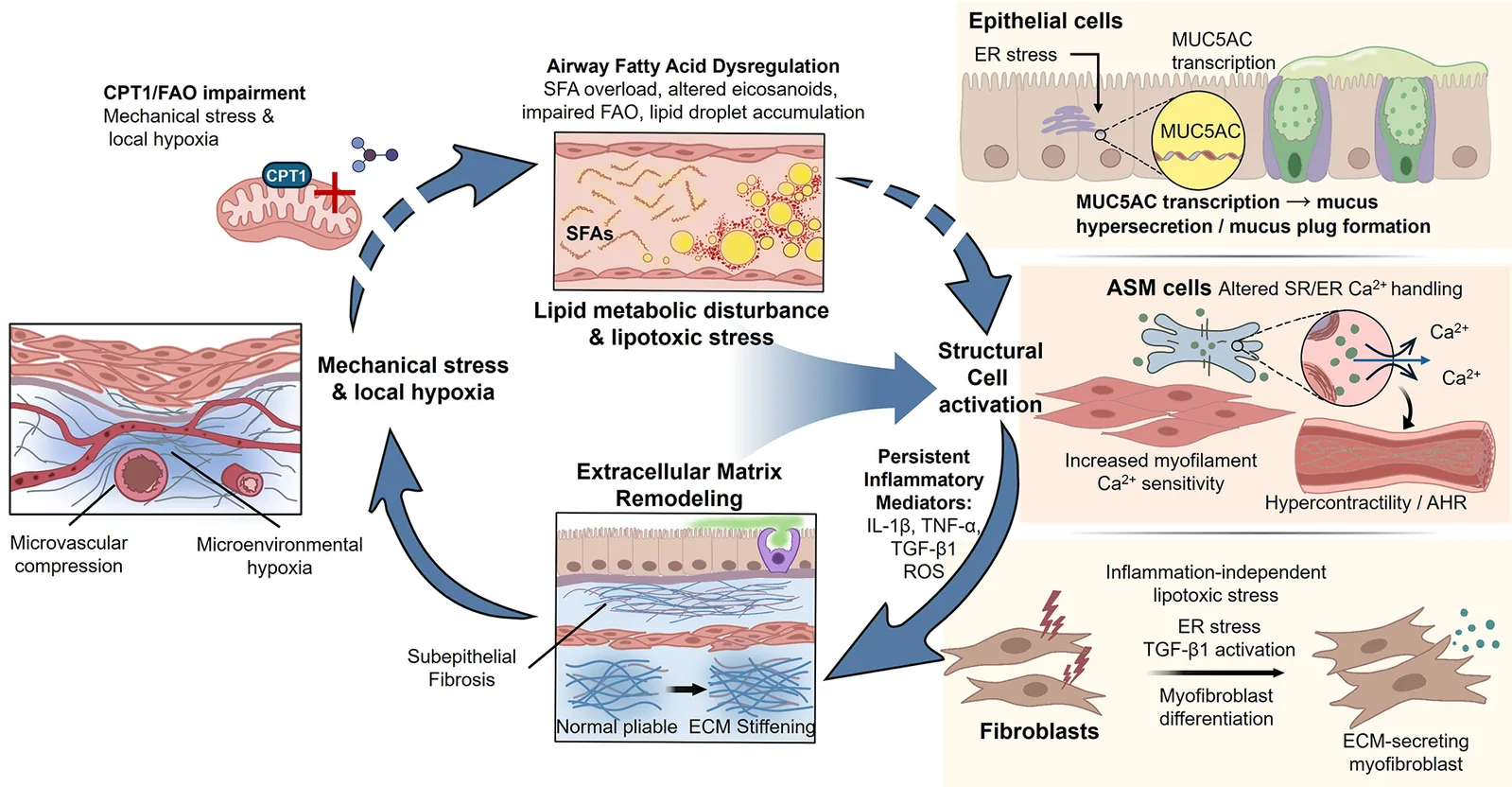
Metabolic–inflammatory–mechanical feedback loop in fatty acid dysregulation-mediated airway remodeling. Airway fatty acid dysregulation, characterized by SFA overload, altered eicosanoid signaling, impaired FAO, and lipid droplet accumulation, promotes lipotoxic stress and structural cell activation. In epithelial cells, ASM cells, and fibroblasts, this process contributes to mucus hypersecretion, AHR, myofibroblast differentiation, and ECM deposition. Progressive ECM stiffening and subepithelial fibrosis further induce mechanical stress, microvascular compression, and local hypoxia, which may impair CPT1-mediated FAO and reinforce lipid metabolic disturbance. This feedback loop links fatty acid dysregulation to persistent airway remodeling and difficult-to-treat asthma. The complete metabolic–inflammatory–mechanical loop is presented as an integrative conceptual framework rather than a fully established causal sequence in asthma.

## 6 Future perspectives and concluding remarks

Targeting fatty acid metabolic dysregulation presents an emerging opportunity for precision medicine in asthma, and may complement conventional anti-inflammatory strategies through phenotype-matched metabolic modulation. Traditional asthma stratification relies primarily on inflammatory biomarkers, which may not fully capture metabolic heterogeneity associated with severe disease, corticosteroid resistance, and obesity-associated phenotypes. Lipidomic studies suggest that distinct fatty acid signatures may provide complementary information for exploratory patient stratification, suggesting that metabolic profiles may represent an additional layer for defining asthma heterogeneity (49). Future research should evaluate whether integrating systemic lipid profiles with airway-specific metabolic measurements can improve metabolic phenotyping and prediction of treatment responses (56). At present, lipidomic biomarkers and related metabolic signatures remain investigational, and further prospective validation is required before they can be incorporated into routine asthma phenotyping or treatment selection, warranting continued investigation in future studies. **Figure 3** summarizes a translational framework in which systemic lipidomic profiling, airway-specific metabolic characterization, and phenotype-based stratification may inform the future development of precision immunometabolic interventions for heterogeneous asthma populations.

**Figure 3:**
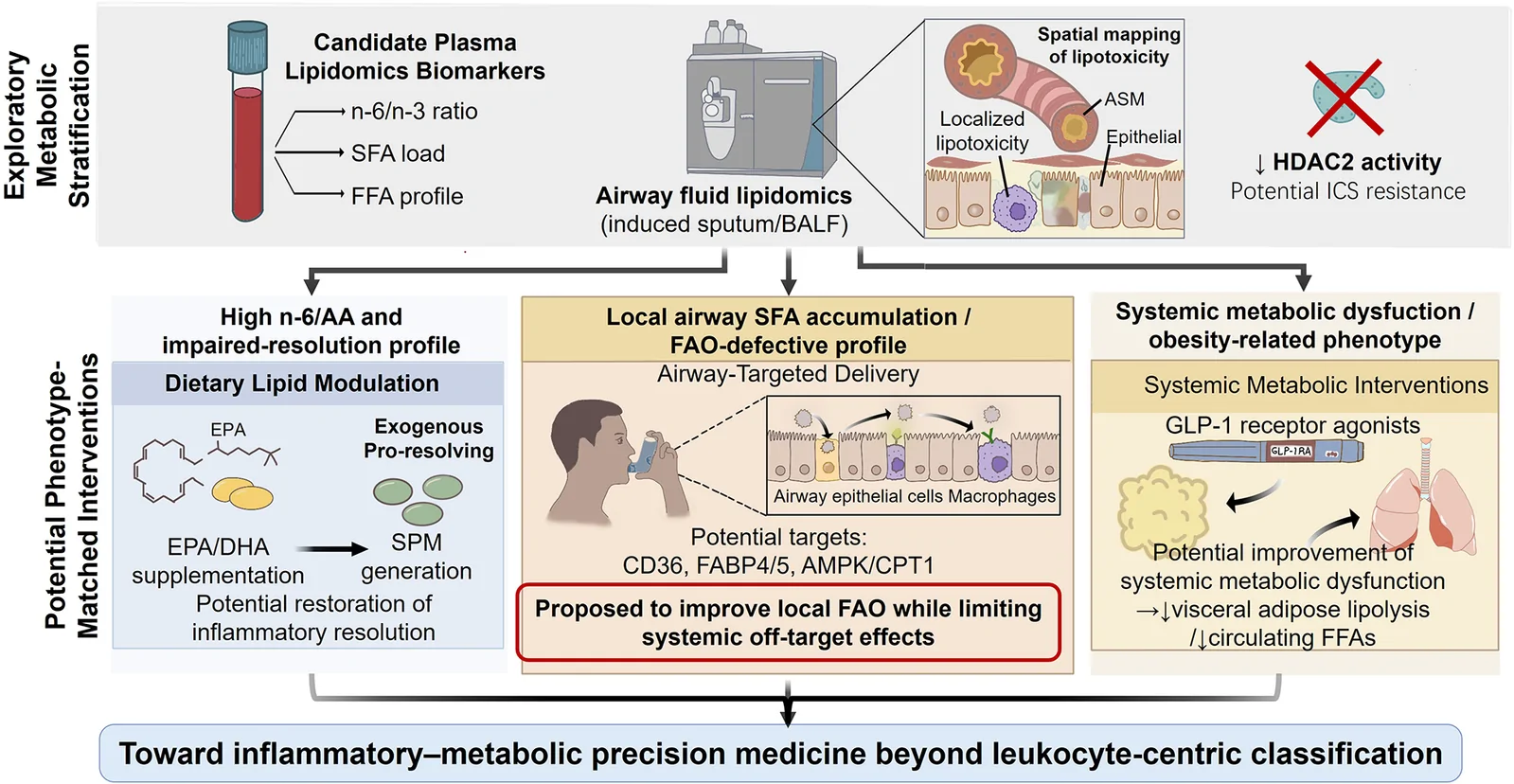
Proposed translational framework for investigational phenotype-matched immunometabolic interventions in asthma. The proposed integrative framework combines plasma lipidomics, airway fluid lipidomics, and spatial mapping of airway lipotoxicity to explore lipid-associated asthma phenotypes and potential markers of ICS resistance. Potential therapeutic strategies are proposed according to dominant metabolic configurations: dietary lipid modulation for high n-6/AA and impaired-resolution phenotypes, airway-targeted delivery for local SFA accumulation or FAO-defective phenotypes, and systemic metabolic interventions for obesity-related metabolic dysfunction. This framework illustrates a potential transition from leukocyte-centric classification toward inflammatory–metabolic precision medicine in asthma.

Metabolic interventions are emerging as potential adjunctive strategies for selected asthma phenotypes characterized by systemic metabolic dysfunction. Emerging metabolic therapies are being investigated for their potential to improve asthma outcomes by targeting adipose tissue dysfunction, circulating free fatty acid excess, and metabolic inflammation (70, 71). Concurrently, airway-targeted metabolic strategies may provide opportunities to directly modulate local metabolic abnormalities while minimizing systemic adverse effects. Future approaches, including inhaled molecules or cell-specific delivery platforms, may enable selective regulation of metabolic pathways in airway immune and structural cells (72, 73).

Translational implementation of metabolic approaches into clinical asthma care requires prospective longitudinal studies to determine whether specific lipid abnormalities represent causal drivers of disease progression or secondary consequences of chronic inflammation. Such studies should also clarify whether T2-high and non-T2 lipid signatures emerge after inflammatory divergence or participate in shaping distinct inflammatory phenotypes. Prospective studies are also needed to establish reproducible biomarker thresholds and to determine whether lipidomic-guided treatment allocation improves clinical outcomes beyond current phenotype-based approaches (49). Integrating single-cell sequencing with spatially resolved metabolic approaches may help define cell-specific metabolic networks and clarify how systemic metabolic disturbances related to localized airway immune responses and structural remodeling (15). Ultimately, if prospectively validated, combining lipidomic biomarkers with clinical phenotyping may enable phenotype-matched interventions that target metabolic vulnerabilities across heterogeneous asthma subtypes.
